# Nomogram for predicting in-hospital mortality in trauma patients undergoing resuscitative endovascular balloon occlusion of the aorta: a retrospective multicenter study

**DOI:** 10.1038/s41598-024-59861-3

**Published:** 2024-04-22

**Authors:** Byungchul Yu, Jayun Cho, Byung Hee Kang, Kyounghwan Kim, Dong Hun Kim, Sung Wook Chang, Pil Young Jung, Yoonjung Heo, Wu Seong Kang

**Affiliations:** 1 Traumatology, Gachon University College of Medicine, Department of Trauma Surgery, Gachon University Gil Medical Center, Incheon, Republic of Korea; 2 Department of Trauma Surgery, Gachon University Gil Medical Center, Incheon, Republic of Korea; 3 Division of Trauma Surgery, Department of Surgery, Ajou School of Medicine, Suwon, Republic of Korea; 4 Department of Trauma Surgery, Jeju Regional Trauma Center, Cheju Halla General Hospital, 65, Doryeong-ro, Jeju-si, Jeju-do Republic of Korea; 5 Division of Trauma Surgery, Department of Surgery, Dankook University College of Medicine, Cheonan, Republic of Korea; 6 Department of Thoracic and Cardiovascular Surgery, Trauma Center, Dankook University Hospital, Cheonan, Republic of Korea; 7 Department of Trauma and Acute Care Surgery, Yonsei University Wonju Severance Christian Hospital, Wonju, Republic of Korea; 8 Department of Trauma Surgery, Trauma Center, Dankook University Hospital, Cheonan, Republic of Korea

**Keywords:** Resuscitative endovascular balloon occlusion of aorta, Hemorrhage, Trauma, Injuries, Diseases, Health care, Medical research, Risk factors

## Abstract

Recently, resuscitative endovascular balloon occlusion of the aorta (REBOA) had been introduced as an innovative procedure for severe hemorrhage in the abdomen or pelvis. We aimed to investigate risk factors associated with mortality after REBOA and construct a model for predicting mortality. This multicenter retrospective study collected data from 251 patients admitted at five regional trauma centers across South Korea from 2015 to 2022. The indications for REBOA included patients experiencing hypovolemic shock due to hemorrhage in the abdomen, pelvis, or lower extremities, and those who were non-responders (systolic blood pressure (SBP) < 90 mmHg) to initial fluid treatment. The primary and secondary outcomes were mortality due to exsanguination and overall mortality, respectively. After feature selection using the least absolute shrinkage and selection operator (LASSO) logistic regression model to minimize overfitting, a multivariate logistic regression (MLR) model and nomogram were constructed. In the MLR model using risk factors selected in the LASSO, five risk factors, including initial heart rate (adjusted odds ratio [aOR], 0.99; 95% confidence interval [CI], 0.98–1.00; p = 0.030), initial Glasgow coma scale (aOR, 0.86; 95% CI 0.80–0.93; p < 0.001), RBC transfusion within 4 h (unit, aOR, 1.12; 95% CI 1.07–1.17; p < 0.001), balloon occlusion type (reference: partial occlusion; total occlusion, aOR, 2.53; 95% CI 1.27–5.02; p = 0.008; partial + total occlusion, aOR, 2.04; 95% CI 0.71–5.86; p = 0.187), and post-REBOA systolic blood pressure (SBP) (aOR, 0.98; 95% CI 0.97–0.99; p < 0.001) were significantly associated with mortality due to exsanguination. The prediction model showed an area under curve, sensitivity, and specificity of 0.855, 73.2%, and 83.6%, respectively. Decision curve analysis showed that the predictive model had increased net benefits across a wide range of threshold probabilities. This study developed a novel intuitive nomogram for predicting mortality in patients undergoing REBOA. Our proposed model exhibited excellent performance and revealed that total occlusion was associated with poor outcomes, with post-REBOA SBP potentially being an effective surrogate measure.

## Introduction

Trauma remains the leading cause of mortality globally and is often exacerbated by uncontrolled hemorrhage^1–3^. Mortality due to severe hemorrhage has always been the primary concern of trauma surgeons given the need for early detection and expeditious bleeding control^2^. The damage control concept including surgery and resuscitation, has reduced hemorrhage-related death in trauma patients. However, patients with severe hemorrhage due to abdominal or pelvic injuries, which often cause massive bleeding, sometimes fail to reach the operation room or angiographic suite for expeditious hemostasis. For these severe patients, conventional surgical treatment, such as thoracotomy and exploratory laparotomy, conducted in the emergency department can promote substantial morbidity and mortality.

In recent years, the introduction of resuscitative endovascular balloon occlusion of the aorta (REBOA) has triggered a significant shift in the approach to managing severe hemorrhage in trauma patients. Traditional hemostatic methods, such as emergency laparotomy, preperitoneal pelvic packing, and angioembolization, have provided effective hemostasis. However, they also have limitations, such as their inability to promptly initiate hemostasis in extremely severe patients. REBOA involves the insertion of a balloon-tipped catheter into the aorta, which, when inflated, occludes blood flow to the injured area and inhibits excessive bleeding in distal organs, such as the abdomen or pelvis^4^. Although REBOA is not a definitive hemostatic procedure, it can preserve cerebral or cardiac blood flow and consequently serve as a bridge to the next hemostatic procedure.

The implementation of REBOA has shown promising outcomes, including effective hemorrhage control and improved hemodynamic stability in various traumatic injuries^4^. However, prospective studies regarding REBOA have been limited, with a recent randomized controlled trial in the UK showing that “REBOA does not reduce, may increase, mortality compared with the non-REBOA protocol”^5^. Various challenges, such as ischemic complications, also necessitate careful patient selection and technique refinement^4^. Therefore, relevant indications for REBOA remain unclear, warranting further studies^6^. Moreover, identifying risk factors for mortality and predicting its occurrence are crucial considering that REBOA is an invasive procedure and has been associated with critical complications, such as limb ischemia and acute kidney injury^4^.

The current study aimed to investigate the risk factors associated with mortality after REBOA and construct a model for predicting mortality.

## Material and methods

### Study design and data sources

All procedures performed in this retrospective observational multicenter study were conducted in accordance with relevant guidelines^7,8^. This study aimed to construct a prediction model for predicting mortality in trauma patients undergoing REBOA. The primary outcome of our study was mortality due to exsanguination, whereas the secondary outcome was overall mortality.

This study was conducted at five regional trauma centers across South Korea (Ajou University Hospital, Cheju Halla General Hospital, Dankook University Hospital, Gachon University Gil Medical Center, and Yonsei University Wonju Severance Christian Hospital), which correspond to level 1 trauma centers in the US. Institutional review board (IRB) approval was obtained from Cheju Halla General Hospital, Ajou University Hospital, Dankook University Hospital, Gachon University Gil Medical Center, and Yonsei University Wonju Severance Christian Hospital (IRB numbers: CHH2023-L16-01, AJOUIRB-DB-2023-524, DKUH 2023-09-003, GCIRB2023-325, and CR323146, respectively). Informed consent was waived by the IRB given the observational nature of this study. All patient data were coded to ensure subject privacy and data confidentiality. In 2015, the REBOA catheter was first introduced in South Korea. Hence, we collected all consecutive cases starting from the first case. In the early stage, we used Reliant™ (compatible with 12 Fr sheath, Medtronic, United States) and Cook Coda™ (compatible to 12 Fr sheath, Cook Medical, United States) balloon catheters. Since the introduction of Rescue Balloon™ (Tokai Medical Products, Aichi, Japan), which is compatible with 7 Fr sheaths, in South Korea by 2018, all trauma centers have used Rescue Balloon™ catheter to minimize catheter-related complications. We collected data for all consecutive trauma patients undergoing REBOA, which include the Injury Severity Score (ISS), Abbreviated Injury Scale (AIS), transfusion, surgical procedures, laboratory findings, morbidities, mortality, results of focused assessment with sonography for trauma (FAST), and other clinical variables.

### Study population, definitions, and inclusion and exclusion criteria

This study enrolled consecutive patients with trauma who visited five trauma centers between December 2015 and December 2021. The indications for REBOA were as follows: (1) patients with hypovolemic shock due to hemorrhage in the abdomen (intraabdominal fluid in the FAST exam), pelvis, or lower extremity and (2) nonresponders (systolic blood pressure (SBP) < 90 mmHg) to initial fluid treatment. The exclusion criteria were as follows: (1) unsuccessful REBOA procedure, including patients who did not undergo balloon inflation after insertion of REBOA catheter (n = 13), and (2) refusal of treatment (n = 1). In-hospital mortality was categorized into five types: mortality due to exsanguination, mortality due to brain injury, mortality due to acute respiratory distress syndrome, mortality due to multiple organ dysfunction syndrome, and mortality due to sepsis. When a patient had two or more causes of mortality, multiple causes of mortality were coded. The balloon occlusion type was categorized as partial or complete. “Complete occlusion” was defined as balloon inflation until the surgeon perceives resistance against the aortic wall during inflation using a syringe.^9^ In contrast, “partial occlusion” was defined as gradual inflation until proximal hemodynamics improves without feeling resistance.^10^ When the target blood pressure was not maintained by partial occlusion, additional inflation, including total occlusion, was conducted, which was defined as “partial + total occlusion.” When total occlusion was performed followed by deflation (partial occlusion), it was also defined as “partial + total occlusion”. The decision between partial or complete occlusion was determined by the discretion of the clinician who conducted the resuscitation and the REBOA procedure. Aortic occlusion level was defined into the following three levels: Zone 1 refers to the area below the origin of the left subclavian artery to above the celiac artery, Zone 2 refers to the area from the celiac artery to the lowest renal artery, and Zone 3 refers to the area below the lowest renal artery and above the aortic bifurcation.

### Statistical analysis

Continuous data were presented as median and interquartile range (IQR), whereas categorical data were presented as proportions. Continuous data were compared using Student’s t-test or Mann–Whitney U test. Proportions were compared using the Chi-square or Fisher’s exact tests as appropriate. Significance was set at p < 0.05. All statistical analyses were conducted using the R language version 4.3.0 (R foundation, Vienna, Austria). We used the “autoReg,” “pROC,” “glmnet,” “tidyverse,” “rms,” and “curves” packages for data analysis and visualization.

To minimize overfitting and enhance the accuracy of the new dataset in our prediction model, we used the least absolute shrinkage and selection operator (LASSO) to shrink the regression coefficients to zero^11,12^. We performed tenfold cross-validation to select an optimal hyperparameter (λ). In the cross-validation, the optimal λ was selected as the most regularized model to keep the error within one standard error of the minimum^11^. Several risk factors for mortality due to exsanguination and overall mortality, which included age, sex, injury mechanism, ISS, AIS (head, chest, abdomen, pelvis, and extremity), initial vital sign, SBP (before and after REBOA procedure), SBP change before and after REBOA, transfusion, main bleeding organ, FAST results, Young–Burgess classification of pelvic fracture, REBOA balloon position, REBOA balloon occlusion type (partial of complete), and surgical procedure (before and after REBOA) were input into the LASSO regression model.

After feature selection using the LASSO regression model, we constructed a multivariable logistic regression (MLR) model. Based on the logistic regression model, we created a nomogram, a graphical calculation device that allows for approximate probability computation^13^. Receiver operator characteristic (ROC) curves were used to evaluate the performance of the prediction model and calculate the area under the ROC curve (AUROC). Youden’s index was used to calculate the optimal cutoff value^14^. To validate our models, a bootstrapping method that replicates the original dataset by 1000 resamples was used to quantify any overfitting^15,16^. Somers’ D was calculated to evaluate model performance. The relationship between Somers’ D and the c-index (AUROC) can been shown as follows: Dxy = 2 (c − 0.5), with Dxy ranging from − 1 to 1^17^. We ran 1000 bootstrap replicates, which was used as the training model. Decision curve analysis was applied to assess the net clinical benefit of the model^18^.

### Institutional Review Board Statement

This study was approved by the institutional review board of the five Hospitals (IRB numbers: CHH2023-L16-01, AJOUIRB-DB-2023-524, DKUH 2023-09-003, GCIRB2023-325, and CR323146, respectively). Informed consent was waived due to the study's observational nature and the de-identification of each patient.

## Results

### Patient characteristics

Table 1 presents the baseline characteristics of the included patients and their comparison according to mortality due to exsanguination and overall mortality. Table 2 presents the comparison of REBOA procedure according to mortality due to exsanguination and overall mortality. Meanwhile, Table 3 summarizes data regarding morbidity and mortality. Throughout the study period, 251 patients who underwent REBOA were included and divided into two groups: those who survived and those who died. Overall, 170 patients (67.7%) died, with 123 (49.0%) patients dying due to exsanguination. Moreover, 21 patients (8.3%) had two or more causes of death. No difference in mortality was observed according to the participating center. Blunt injury was the most common mechanism cause of mortality (96.4%). The overall morbidity was 57.0%.

**Table 1:** Baseline characteristics of the patients and comparison between patients who did and did not survive REBOA.

Variable	Stats	N	Missing	Missing rate	Mortality due to exsanguination			Overall mortality		
					Survived	Died	p	Survived	Died	p
					(n = 128)	(n = 123)		(n = 81)	(n = 170)	
Hospital		251	0	(0.0%)			0.295			0.991
Ajou University Hospital	45 (17.9%)				25 (19.5%)	20 (16.3%)		15 (18.5%)	30 (17.6%)	
Cheju Halla General Hospital	24 (9.6%)				14 (10.9%)	10 (8.1%)		8 (9.9%)	16 (9.4%)	
Dankook University Hospital	96 (38.2%)				50 (39.1%)	46 (37.4%)		31 (38.3%)	65 (38.2%)	
Gachon University Gil Medical Center	51 (20.3%)				27 (21.1%)	24 (19.5%)		17 (21.0%)	34 (20.0%)	
Yonsei University Wonju Severance Christian Hospital	35 (13.9%)				12 (9.4%)	23 (18.7%)		10 (12.3%)	25 (14.7%)	
Age	53.0 [38.0;66.0]	251	0	(0.0%)	53.0 [38.5;66.0]	52.0 [35.5;66.0]	0.779	49.0 [37.0;61.0]	54.5 [38.0;69.0]	0.151
Sex		251	0	(0.0%)			0.815			0.458
F	68 (27.1%)				36 (28.1%)	32 (26.0%)		19 (23.5%)	49 (28.8%)	
M	183 (72.9%)				92 (71.9%)	91 (74.0%)		62 (76.5%)	121 (71.2%)	
Hospital stay	2.0 [1.0;25.5]	251	0	(0.0%)	24.5 [8.0;56.0]	1.0 [1.0; 2.0]	< 0.001	41.0 [23.0;71.0]	1.0 [1.0; 2.0]	< 0.001
Injury mechanism		251	0	(0.0%)			0.139			0.398
Blunt injury	242 (96.4%)				122 (95.3%)	120 (97.6%)		77 (95.1%)	165 (97.1%)	
Penetrating injury	7 (2.8%)				6 (4.7%)	1 (0.8%)		4 (4.9%)	3 (1.8%)	
Blunt + penetrating injury	1 (0.4%)				0 (0.0%)	1 (0.8%)		0 (0.0%)	1 (0.6%)	
Crushing injury	1 (0.4%)				0 (0.0%)	1 (0.8%)		0 (0.0%)	1 (0.6%)	
Initial SBP (mmHg)	60.0 [0.0;82.5]	251	0	(0.0%)	69.0 [56.0;90.0]	40.0 [0.0;75.0]	< 0.001	79.0 [60.0;95.0]	52.0 [0.0;75.0]	< 0.001
Initial HR (beat/min)	98.0 [62.0;121.0]	251	0	(0.0%)	103.0 [86.0;123.0]	83.0 [0.0;118.5]	0.000	104.0 [89.0;123.0]	91.0 [0.0;120.0]	0.001
Initial RR (rate/min)	18.0 [0.0;24.0]	251	0	(0.0%)	20.0 [16.0;24.0]	8.0 [0.0;21.5]	< 0.001	21.0 [18.0;24.0]	11.5 [0.0;23.0]	< 0.001
Initial body temperature (°C)	36.0 [35.0;36.3]	238	13	(5.2%)	36.0 [35.4;36.3]	35.7 [34.5;36.1]	0.003	36.1 [35.8;36.3]	35.6 [34.6;36.1]	< 0.001
Initial GCS	6.0 [3.0;13.0]	251	0	(0.0%)	11.0 [5.0;15.0]	3.0 [3.0; 7.0]	< 0.001	13.0 [8.0;15.0]	3.0 [3.0; 9.0]	< 0.001
ISS	34.0 [25.0;43.0]	251	0	(0.0%)	34.0 [22.0;43.0]	34.0 [26.0;41.0]	0.790	29.0 [18.0;43.0]	34.0 [26.0;43.0]	0.035
AIS-head	0.0 [0.0;3.0]	251	0	(0.0%)	0.0 [0.0; 3.0]	0.0 [0.0; 1.5]	0.004	0.0 [0.0; 2.0]	0.0 [0.0; 3.0]	0.571
AIS-chest	3.0 [0.0;3.0]	251	0	(0.0%)	3.0 [0.0; 3.0]	3.0 [0.0; 3.0]	0.190	3.0 [0.0; 3.0]	3.0 [0.0; 3.0]	0.053
AIS-abdomen	3.0 [2.0;4.0]	251	0	(0.0%)	3.0 [2.0; 4.0]	4.0 [2.0; 4.0]	0.066	3.0 [2.0; 4.0]	3.0 [2.0; 4.0]	0.849
AIS extremity and pelvis	3.0 [0.0;5.0]	251	0	(0.0%)	3.0 [0.0; 5.0]	3.0 [0.0; 5.0]	0.690	3.0 [0.0; 5.0]	3.0 [0.0; 5.0]	0.946
Transfusion										
Door-to-transfusion time (min)	17.0 [11.0;25.0]	251	0	(0.0%)	17.0 [12.0;24.5]	16.0 [10.0;25.0]	0.376	17.0 [11.0;24.0]	16.5 [11.0;26.0]	0.704
RBC transfusion within 4 h (unit)	13.0 [9.0;19.0]	251	0	(0.0%)	11.0 [8.0;15.5]	16.0 [10.0;23.0]	< 0.001	11.0 [9.0;15.0]	15.0 [10.0;22.0]	< 0.001
FFP transfusion within 4 h (unit)	8.0 [4.0;12.0]	251	0	(0.0%)	7.0 [4.0;10.0]	9.0 [4.0;13.0]	0.024	6.0 [4.0; 9.0]	8.0 [4.0;13.0]	0.014
Platelet transfusion within 4 h (unit)	0.0 [0.0;0.0]	251	0	(0.0%)	0.0 [0.0; 0.0]	0.0 [0.0; 0.0]	0.792	0.0 [0.0; 0.0]	0.0 [0.0; 0.0]	0.898
RBC transfusion within 24 h (unit)	5.0 [0.0;15.0]	251	0	(0.0%)	17.0 [11.0;30.5]	21.0 [12.0;36.0]	0.028	15.0 [11.0;24.0]	21.5 [13.0;37.0]	0.001
FFP transfusion within 24 h (unit)	5.0 [0.0;12.0]	251	0	(0.0%)	13.0 [8.0;21.5]	13.0 [6.0;25.0]	0.804	12.0 [8.0;18.0]	14.5 [7.0;27.0]	0.120
Platelet transfusion within 24 h (unit)	0.0 [0.0;10.0]	251	0	(0.0%)	8.0 [0.0;10.0]	0.0 [0.0; 1.0]	< 0.001	8.0 [0.0;10.0]	0.0 [0.0; 9.0]	0.003
Bleeder										
Liver	57 (22.7%)	251	0	(0.0%)	20 (15.6%)	37 (30.1%)	0.010	15 (18.5%)	42 (24.7%)	0.351
Spleen	31 (12.4%)	251	0	(0.0%)	16 (12.5%)	15 (12.2%)	1.000	11 (13.6%)	20 (11.8%)	0.839
Mesentery	58 (23.1%)	251	0	(0.0%)	33 (25.8%)	25 (20.3%)	0.381	27 (33.3%)	31 (18.2%)	0.013
Pelvis	104 (41.4%)	251	0	(0.0%)	51 (39.8%)	53 (43.1%)	0.694	28 (34.6%)	76 (44.7%)	0.165
Kidney	16 (6.4%)	251	0	(0.0%)	6 (4.7%)	10 (8.1%)	0.391	3 (3.7%)	13 (7.6%)	0.358
Retroperitoneum	17 (6.8%)	251	0	(0.0%)	11 (8.6%)	6 (4.9%)	0.305	6 (7.4%)	11 (6.5%)	0.333
Lung	23 (9.2%)	251	0	(0.0%)	6 (4.7%)	17 (13.8%)	0.022	3 (3.7%)	20 (11.8%)	0.066
Brain hemorrhage	13 (5.2%)	251	0	(0.0%)	10 (7.8%)	3 (2.4%)	0.102	1 (1.2%)	12 (7.1%)	0.101
Major vessel	11 (4.4%)	251	0	(0.0%)	3 (2.3%)	8 (6.5%)	0.193	1 (1.2%)	10 (5.9%)	0.176
Extremity	17 (6.8%)	251	0	(0.0%)	11 (8.6%)	6 (4.9%)	0.358	6 (7.4%)	11 (6.5%)	0.994
Pancreas	1 (0.4%)	251	0	(0.0%)	0 (0.0%)	1 (0.8%)	0.984	0 (0.0%)	1 (0.6%)	1.000
Spine	1 (0.4%)	251	0	(0.0%)	1 (0.8%)	0 (0.0%)	1.000	0 (0.0%)	1 (0.6%)	1.000
Young Burgess classification		251	0	(0.0%)			0.682			0.218
No pelvic fracture	126 (50.2%)				65 (50.8%)	61 (49.6%)		48 (59.3%)	78 (45.9%)	
Anteroposterior compression	35 (13.9%)				17 (13.3%)	18 (14.6%)		10 (12.3%)	25 (14.7%)	
Lateral compression	71 (28.3%)				34 (26.6%)	37 (30.1%)		17 (21.0%)	54 (31.8%)	
Vertical shear	19 (7.6%)				12 (9.4%)	7 (5.7%)		6 (7.4%)	13 (7.6%)	
FAST finding		251	0	(0.0%)			0.015			0.163
Negative	80 (31.9%)				50 (39.1%)	30 (24.4%)		25 (30.9%)	55 (32.4%)	
Positive in the abdomen	137 (54.6%)				62 (48.4%)	75 (61.0%)		49 (60.5%)	88 (51.8%)	
Positive in the chest	11 (4.4%)				8 (6.2%)	3 (2.4%)		3 (3.7%)	8 (4.7%)	
Positive in the abdomen and chest	22 (8.8%)				7 (5.5%)	15 (12.2%)		3 (3.7%)	19 (11.2%)	

Values are presented as number (%) or median (interquartile range). REBOA, resuscitative endovascular balloon occlusion of aorta; SBP, systolic blood pressure; HR, heart rate; RR, respiratory rate; GCS, Glasgow coma scale; ISS, injury severity score; AIS, abbreviated injury scale;; RBC, red blood cell; FFP, fresh frozen plasma; FAST, focused assessment with sonography in trauma;

**Table 2:** Comparison of REBOA procedure between patients who did and did not survive REBOA.

Variable	Stats	N	Missing	Missing rate	Mortality due to exsanguination			Overall mortality		
					Survived	Died	p	Survived	Died	p
					(n = 128)	(n = 123)		(n = 81)	(n = 170)	
REBOA device		251	0	(0.0%)			0.393			0.394
Rescue™	202 (80.5%)				107 (83.6%)	95 (77.2%)		69 (85.2%)	133 (78.2%)	
Relient™	39 (15.5%)				16 (12.5%)	23 (18.7%)		9 (11.1%)	30 (17.6%)	
Coda™	10 (4.0%)				5 (3.9%)	5 (4.1%)		3 (3.7%)	7 (4.1%)	
Puncture site		251	0	(0.0%)			0.779			0.886
Right femoral artery	158 (62.9%)				79 (61.7%)	79 (64.2%)		52 (64.2%)	106 (62.4%)	
Left femoral artery	93 (37.1%)				49 (38.3%)	44 (35.8%)		29 (35.8%)	64 (37.6%)	
Access method		251	0	(0.0%)			0.054			0.356
Blind	222 (88.4%)				119 (93.0%)	103 (83.7%)		75 (92.6%)	147 (86.5%)	
Sono-guided	23 (9.2%)				8 (6.2%)	15 (12.2%)		5 (6.2%)	18 (10.6%)	
Open method	6 (2.4%)				1 (0.8%)	5 (4.1%)		1 (1.2%)	5 (2.9%)	
Door-to-puncture time (min)	23.0 [15.0;38.0]	166	85	(33.9%)	23.5 [16.0;38.0]	22.0 [14.5;38.5]	0.584	23.0 [16.0;42.0]	23.0 [15.0;36.5]	0.813
Door-to-balloon time (min)	33.0 [22.0;51.0]	237	14	(5.6%)	34.0 [22.0;54.0]	33.0 [21.5;49.0]	0.364	35.0 [22.0;59.0]	33.0 [22.0;47.0]	0.356
Puncture-to-balloon time (min)	2.0 [0.0;7.0]	165	86	(34.3%)	2.0 [0.0; 6.0]	3.0 [0.0; 8.0]	0.979	2.0 [0.0; 5.0]	3.0 [0.0; 7.5]	1.000
Balloon position		251	0	(0.0%)			0.055			0.368
Zone 1	154 (61.4%)				73 (57.0%)	81 (65.9%)		49 (60.5%)	105 (61.8%)	
Zone 2	4 (1.6%)				2 (1.6%)	2 (1.6%)		0 (0.0%)	4 (2.4%)	
Zone 3	81 (32.3%)				50 (39.1%)	31 (25.2%)		30 (37.0%)	51 (30.0%)	
Zone 1 → Zone 3	8 (3.2%)				1 (0.8%)	7 (5.7%)		1 (1.2%)	7 (4.1%)	
Zone 3 → Zone 1	4 (1.6%)				2 (1.6%)	2 (1.6%)		1 (1.2%)	3 (1.8%)	
Occlusion type		251	0	(0.0%)			< 0.001			0.004
Partial occlusion	120 (47.8%)				78 (60.9%)	42 (34.1%)		51 (63.0%)	69 (40.6%)	
Complete occlusion	103 (41.0%)				39 (30.5%)	64 (52.0%)		24 (29.6%)	79 (46.5%)	
Partial + Complete occlusion	28 (11.2%)				11 (8.6%)	17 (13.8%)		6 (7.4%)	22 (12.9%)	
Ballooning volume (cc)	15.0 [10.0;20.0]	116	135	(53.8%)	10.0 [9.0;20.0]	17.0 [10.0;24.5]	0.006	10.0 [8.0;20.0]	15.0 [10.0;20.0]	0.109
Total occlusion time (min)	87.0 [50.0;173.0]	201	50	(19.9%)	78.0 [46.5;137.5]	118.5 [57.0;205.0]	0.011	76.0 [42.0;104.0]	111.5 [56.0;193.0]	0.001
Pre-REBOA SBP (mmHg)	56.0 [43.0;68.0]	251	0	(0.0%)	60.0 [49.5;71.5]	50.0 [0.0;60.0]	< 0.001	65.0 [52.0;75.0]	52.0 [30.0;61.0]	< 0.001
Post-REBOA SBP (mmHg)	94.0 [76.0;113.5]	251	0	(0.0%)	100.5 [88.0;120.0]	83.0 [53.5;104.0]	< 0.001	106.0 [92.0;126.0]	87.5 [64.0;107.0]	< 0.001
SBP change after REBOA (mmHg)	37.0 [20.0;57.0]	251	0	(0.0%)	40.5 [25.0;58.0]	31.0 [12.5;55.5]	0.014	43.0 [25.0;58.0]	34.0 [14.0;56.0]	0.017
Treatment flow		251	0	(0.0%)			0.002			0.003
REBOA → emergency laparotomy	125 (49.8%)				66 (51.6%)	59 (48.0%)		47 (58.0%)	78 (45.9%)	
REBOA → PPP	19 (7.6%)				6 (4.7%)	13 (10.6%)		3 (3.7%)	16 (9.4%)	
REBOA → angiography	31 (12.4%)				22 (17.2%)	9 (7.3%)		14 (17.3%)	17 (10.0%)	
REBOA → PPP → angiography	33 (13.1%)				19 (14.8%)	14 (11.4%)		11 (13.6%)	22 (12.9%)	
REBOA → angiography → PPP	2 (0.8%)				0 (0.0%)	2 (1.6%)		0 (0.0%)	2 (1.2%)	
PPP/angiography/emergency laparotomy → REBOA	9 (3.6%)				6 (4.7%)	3 (2.4%)		4 (4.9%)	5 (2.9%)	
REBOA → ECMO	1 (0.4%)				1 (0.8%)	0 (0.0%)		0 (0.0%)	1 (0.6%)	
Intraoperative REBOA	2 (0.8%)				2 (1.6%)	0 (0.0%)		2 (2.5%)	0 (0.0%)	
EDT → REBOA	4 (1.6%)				0 (0.0%)	4 (3.3%)		0 (0.0%)	4 (2.4%)	
No additional procedure after REBOA	3 (1.2%)				2 (1.6%)	1 (0.8%)		0 (0.0%)	3 (1.8%)	
No response to REBOA and no additional procedures	22 (8.8%)				4 (3.1%)	18 (14.6%)		0 (0.0%)	22 (12.9%)	
Single procedure following REBOA	175 (69.7%)	251	0	(0.0%)	94 (73.4%)	81 (65.9%)	0.242	64 (79.0%)	111 (65.3%)	0.039
Two procedures following REBOA	35 (13.9%)	251	0	(0.0%)	19 (14.8%)	16 (13.0%)	0.812	11 (13.6%)	24 (14.1%)	1.000
REBOA during CPR	43 (17.1%)	251	0	(0.0%)	3 (2.3%)	40 (32.5%)	< 0.001	0 (0.0%)	43 (25.3%)	< 0.001

Values are presented as number (%) or median (interquartile range). REBOA, resuscitative endovascular balloon occlusion of aorta; SBP, systolic blood pressure; PPP, preperitoneal pelvic packing; ECMO, extracorporeal membrane oxygenation; EDT, emergency department thoracotomy; CPR, cardiopulmonary resuscitation.

**Table 3:** Comparison of morbidity and mortality between patients who did and did not survive after REBOA.

Variable	Stats	N	Missing	Missing rate	Mortality due to exsanguination			Overall mortality		
					Survived	Died	p	Survived	Died	p
					(n = 128)	(n = 123)		(n = 81)	(n = 170)	
ICU stay (day)	2.0 [1.0;8.0]	251	0	(0.0%)	7.5 [3.0;17.0]	1.0 [1.0; 2.0]	< 0.001	10.0 [4.0;18.0]	1.0 [1.0; 2.0]	< 0.001
Duration of mechanical ventilation (day)	2.0 [1.0;4.0]	251	0	(0.0%)	4.0 [1.0; 8.0]	1.0 [1.0; 2.0]	< 0.001	5.0 [2.0; 8.0]	1.0 [1.0; 2.0]	< 0.001
Morbidity	143 (57.0%)	251	0	(0.0%)	85 (66.4%)	58 (47.2%)	0.003	50 (61.7%)	93 (54.7%)	0.361
Postoperative bleeding	16 (6.4%)	251	0	(0.0%)	9 (7.0%)	7 (5.7%)	0.860	6 (7.4%)	10 (5.9%)	0.852
Wound-related complications	24 (9.6%)	251	0	(0.0%)	24 (18.8%)	0 (0.0%)	< 0.001	15 (18.5%)	9 (5.3%)	0.002
Acute cholecystitis	3 (1.2%)	251	0	(0.0%)	3 (2.3%)	0 (0.0%)	0.260	1 (1.2%)	2 (1.2%)	1.000
Gastrointestinal bleeding	1 (0.4%)	251	0	(0.0%)	1 (0.8%)	0 (0.0%)	1.000	0 (0.0%)	1 (0.6%)	1.000
Intestinal ischemia	1 (0.4%)	251	0	(0.0%)	1 (0.8%)	0 (0.0%)	1.000	0 (0.0%)	1 (0.6%)	1.000
Hepatic failure	2 (0.8%)	251	0	(0.0%)	2 (1.6%)	0 (0.0%)	0.495	0 (0.0%)	2 (1.2%)	0.825
ARDS	19 (7.6%)	251	0	(0.0%)	18 (14.1%)	1 (0.8%)	< 0.001	9 (11.1%)	10 (5.9%)	0.227
Pneumonia	18 (7.2%)	251	0	(0.0%)	18 (14.1%)	0 (0.0%)	< 0.001	12 (14.8%)	6 (3.5%)	0.003
Acute Kidney Injury	72 (28.7%)	251	0	(0.0%)	50 (39.1%)	22 (17.9%)	< 0.001	21 (25.9%)	51 (30.0%)	0.605
Urinary tract infection	5 (2.0%)	251	0	(0.0%)	5 (3.9%)	0 (0.0%)	0.078	4 (4.9%)	1 (0.6%)	0.068
Ileus	3 (1.2%)	251	0	(0.0%)	3 (2.3%)	0 (0.0%)	0.260	3 (3.7%)	0 (0.0%)	0.057
Pulmonary thromboembolism	1 (0.4%)	251	0	(0.0%)	1 (0.8%)	0 (0.0%)	1.000	1 (1.2%)	0 (0.0%)	0.704
Deep vein thrombosis	2 (0.8%)	251	0	(0.0%)	2 (1.6%)	0 (0.0%)	0.495	2 (2.5%)	0 (0.0%)	0.194
Intraabdominal abscess	10 (4.0%)	251	0	(0.0%)	10 (7.8%)	0 (0.0%)	0.005	7 (8.6%)	3 (1.8%)	0.024
Aanstomostic leakage	3 (1.2%)	251	0	(0.0%)	3 (2.3%)	0 (0.0%)	0.260	2 (2.5%)	1 (0.6%)	0.509
CRRT in the ICU	41 (16.3%)	251	0	(0.0%)	30 (23.4%)	11 (8.9%)	0.003	10 (12.3%)	31 (18.2%)	0.319
REBOA-related complication										
No complications	227 (90.4%)	251	0	(0.0%)	111 (86.7%)	116 (94.3%)	0.067	72 (88.9%)	155 (91.2%)	0.728
Puncture vessel injury	5 (2.0%)	251	0	(0.0%)	5 (3.9%)	0 (0.0%)	0.078	3 (3.7%)	2 (1.2%)	0.392
Unexpected balloon migration	3 (1.2%)	251	0	(0.0%)	0 (0.0%)	3 (2.4%)	0.231	0 (0.0%)	3 (1.8%)	0.561
Bowel ischemia	4 (1.6%)	251	0	(0.0%)	2 (1.6%)	2 (1.6%)	1.000	1 (1.2%)	3 (1.8%)	1.000
Skin necrosis	2 (0.8%)	251	0	(0.0%)	2 (1.6%)	0 (0.0%)	0.495	1 (1.2%)	1 (0.6%)	1.000
Extremity necrosis	4 (1.6%)	251	0	(0.0%)	4 (3.1%)	0 (0.0%)	0.141	2 (2.5%)	2 (1.2%)	0.822
Mortality										
Overall	170 (67.7%)	251	0	(0.0%)	47 (36.7%)	123 (100.0%)	< 0.001			
Mortality due to exsanguination	123 (49.0%)	251	0	(0.0%)				0 (0.0%)	123 (72.4%)	< 0.001
Mortality due to brain injury	26 (10.4%)	251	0	(0.0%)	19 (14.8%)	7 (5.7%)	0.030	0 (0.0%)	26 (15.3%)	< 0.001
Mortality due to ARDS	4 (1.6%)	251	0	(0.0%)	4 (3.1%)	0 (0.0%)	0.141	0 (0.0%)	4 (2.4%)	0.394
Mortality due to MODS	19 (7.6%)	251	0	(0.0%)	13 (10.2%)	6 (4.9%)	0.180	0 (0.0%)	19 (11.2%)	0.004
Mortality due to sepsis	11 (4.4%)	251	0	(0.0%)	11 (8.6%)	0 (0.0%)	0.003	0 (0.0%)	11 (6.5%)	0.044
Others	5 (2.0%)	251	0	(0.0%)	4 (3.1%)	1 (0.8%)	0.391	0 (0.0%)	5 (2.9%)	0.282
Mortality within 24 h	121 (48.2%)	251	0	(0.0%)	15 (11.7%)	106 (86.2%)	< 0.001	0 (0.0%)	121 (71.2%)	< 0.001
Unintended insertion into the IVC	3 (1.2%)	251	0	(0.0%)	1 (0.8%)	2 (1.6%)	0.972	0 (0.0%)	3 (1.8%)	0.561

Values are presented as number (%) or median (interquartile range). REBOA, resuscitative endovascular balloon occlusion of the aorta; ICU, intensive care unit; ARDS, Acute respiratory distress syndrome; MODS, Multiple organ dysfunction syndrome; CRRT, continuous renal replacement therapy; IVC, inferior vena cava.

### Risk factor selection using the LASSO logistic regression model

**Figure 1:**
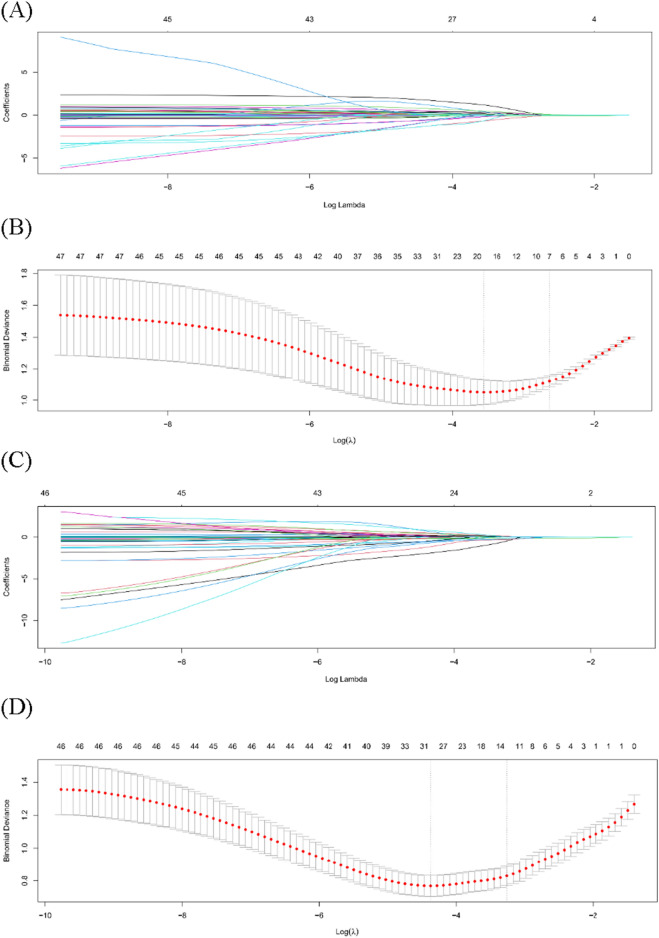
Clinical variables were selected using the LASSO logistic regression model. (**A**) In terms of mortality due to exsanguination, shrinkage of coefficients by hyperparameter (λ). (**B**) In terms of mortality due to exsanguination, hyperparameter selection (λ) using cross-validation. (**C**) In terms of overall mortality, shrinkage of coefficients by hyperparameter (λ). (**D**) In terms of overall mortality, hyperparameter selection (λ) using cross-validation. The dotted line indicates the value of the harmonic log (λ) when the model error is minimized.

Figure 1 presents the results for the LASSO logistic regression model. Figure 1A depicts the shrinkage of coefficients using the hyperparameter (λ), whereas Fig. 1B depicts the model’s accuracy via cross-validation in the mortality due to exsanguination model. Figure 1C depicts the shrinkage of coefficients using the hyperparameter (λ), whereas Fig. 1D depicts the model’s accuracy via cross-validation in the overall mortality model. LASSO shrank the coefficient estimates of the other risk factors toward zero. In the cross-validation, the optimal log (λ) was − 2.6281 and − 3.2563 in the mortality due to exsanguination and overall mortality models, respectively. In terms of mortality due to exsanguination, the LASSO identified seven risk factors, including initial SBP, initial heart rate (HR), initial Glasgow coma scale (GCS), red blood cell (RBC) transfusion within 4 h, balloon occlusion type, pre-REBOA SBP, and post-REBOA SBP. In terms of overall mortality, the LASSO identified 12 risk factors, including age, sex, initial SBP, initial GCS, RBC transfusion within 4 h, mesenteric bleeding, retroperitoneal bleeding, Young–Burgess classification of pelvic fracture, FAST (positive in chest), balloon occlusion type, pre-REBOA-SBP, and post-REBOA-SBP.

### Prediction model and nomogram

**Figure 2:**
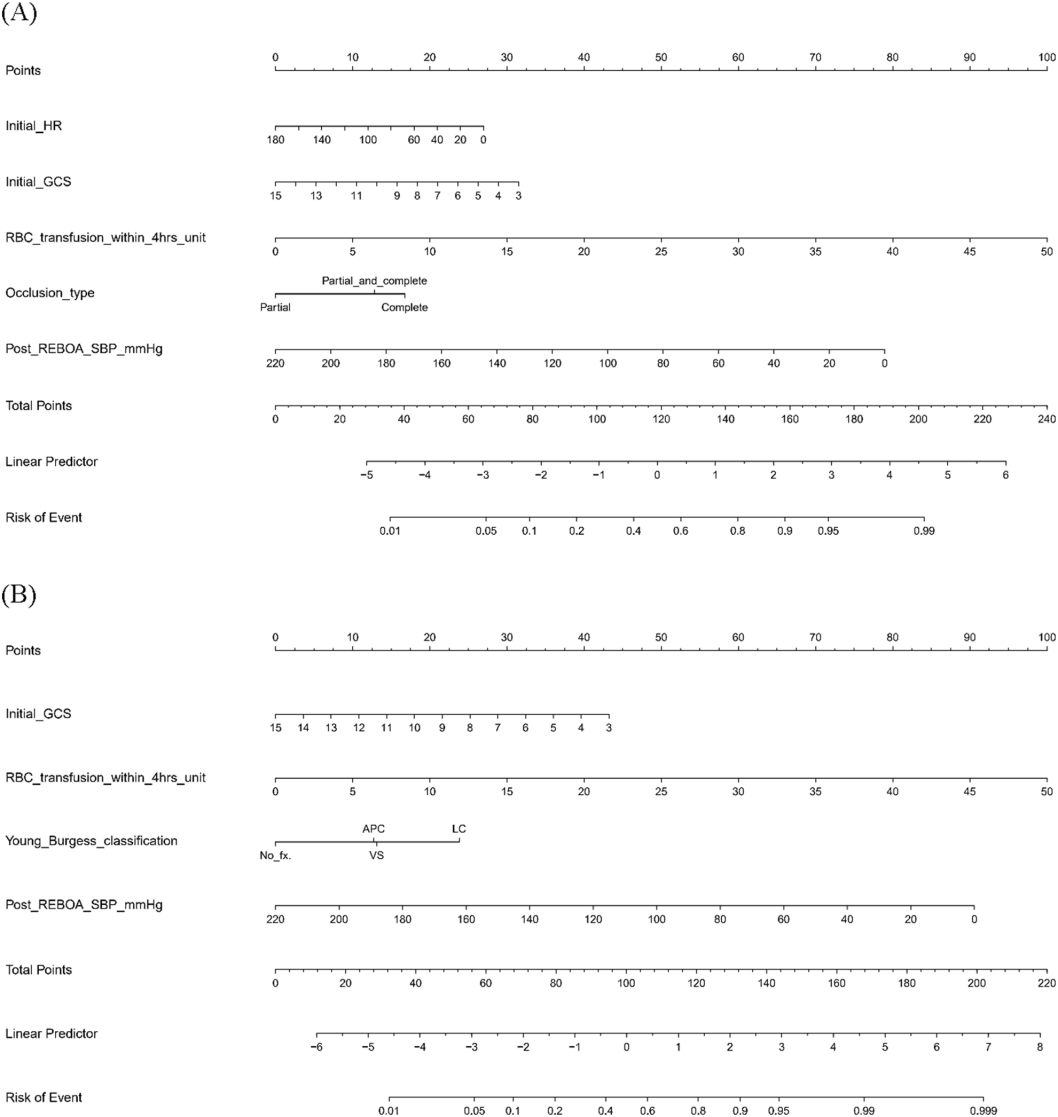
The nomogram predicts the risk of mortality due to exsanguination (**A**) and overall mortality (**B**). Each variable is assigned a score on each axis. The sum of all points for all variables is computed and denoted as the total points. The predicted probability can be obtained on the lowest row corresponding to the sum of total points.

Table 4 summarizes the MLR model using risk factors selected in the LASSO for each model. We constructed a nomogram that predicted exsanguination and overall mortality using significant risk factors based on the MLR (Fig. 2). In the mortality due to exsanguination model, the MLR identified five significant risk factors, namely initial HR (0.99 adjusted odds ratio (aOR), 0.98–1.00 95% confidence interval (CI); p = 0.030), initial GCS (0.86 aOR, 0.80–0.93 95% CI; p < 0.001), RBC transfusion within 4 h (unit, 1.12 aOR, 1.07–1.17 95% CI; p < 0.001), balloon occlusion type (reference: partial occlusion; total occlusion, 2.53 aOR, 1.27–5.02 95% CI; p = 0.008; partial + total occlusion, 2.04 aOR, 0.71–5.86 95% CI; p = 0.187), and post-REBOA SBP (0.98 aOR, 0.97–0.99 95% CI; p < 0.001). In the overall mortality model, the MLR identified four significant risk factors, namely initial GCS (0.81 aOR, 0.74–0.88 95% CI; p < 0.001), RBC transfusion within 4 h (unit, 1.15 aOR, 1.08–1.23 95% CI; p < 0.001), Young–Burgess classification (reference: no pelvic fracture, antero-posterior compression type, 2.91 aOR, 0.93–9.10 95% CI; p = 0.066; lateral compression type, 5.33 aOR, 2.00–14.15 95% CI; p < 0.001; vertical shear type, 1.87 aOR, 0.47–7.51 95% CI; p = 0.375), and post-REBOA SBP (0.98 aOR, 0.96–0.99 95% CI; p = 0.006).

**Table 4:** Multivariate logistic regression model using the risk factors selected by LASSO.

Variable	cOR (univariable)	aOR (multivariable)
Model for predicting mortality due to exsanguination after REBOA		
Initial SBP (mmHg)	0.98 (0.98–0.99; p < 0.001)	
Initial HR (rate/min)	0.99 (0.98–0.99; p < 0.001)	0.99 (0.98–1.00; p = 0.030)
Initial GCS	0.82 (0.77–0.87; p < 0.001)	0.86 (0.80–0.93; p < 0.001)
RBC transfusion within 4 h (unit)	1.08 (1.04–1.12; p < 0.001)	1.12 (1.07–1.17; p < 0.001)
REBOA balloon occlusion type		
Partial occlusion (reference)		
Total occlusion	3.05 (1.76–5.27; p < 0.001)	2.53 (1.27–5.02; p = 0.008)
Partial + total occlusion	2.87 (1.23–6.69; p = 0.015)	2.04 (0.71–5.86; p = 0.187)
Pre-REBOA SBP (mmHg)	0.97 (0.96–0.98; p < 0.001)	
Post-REBOA SBP (mmHg)	0.98 (0.97–0.99; p < 0.001)	0.98 (0.97–0.99; p < 0.001)
Model for predicting overall mortality after the REBOA procedure		
Age (year)	1.01 (1.00–1.02; p = 0.205)	
Sex		
Female (reference)		
Male	0.76 (0.41–1.40; p = 0.372)	
Initial SBP (mmHg)	0.98 (0.97–0.99; p < 0.001)	0.99 (0.98–1.00; p = 0.071)
Initial GCS	0.77 (0.72–0.83; p < 0.001)	0.81 (0.74–0.88; p < 0.001)
RBC transfusion within 4 h (unit)	1.09 (1.05–1.14; p < 0.001)	1.15 (1.08–1.23; p < 0.001)
Bleeding: mesentery		
No (reference)		
Yes	0.45 (0.24–0.82; p = 0.009)	
Bleeding: pelvis		
No (reference)		
Yes	1.53 (0.88–2.65; p = 0.128)	
Bleeding: retroperitoneum		
No (reference)		
Yes	0.85 (0.30–2.40; p = 0.763)	
Young–Burgess classification		
No pelvic fracture (reference)		
Antero–posterior compression	1.54 (0.68–3.48; p = 0.301)	2.91 (0.93–9.10; p = 0.066)
Lateral compression	1.95 (1.02–3.76; p = 0.044)	5.33 (2.00–14.15; p < 0.001)
Vertical shear	1.33 (0.48–3.74; p = 0.585)	1.87 (0.47–7.51; p = 0.375)
FAST		
Negative in the chest (reference)		
Positive in the chest	2.36 (0.93–5.97; p = 0.070)	3.31 (0.88–12.51; p = 0.077)
REBOA balloon occlusion type		
Partial occlusion (reference)		
Total occlusion	2.43 (1.36–4.36; p = 0.003)	
Partial + total occlusion	2.71 (1.02–7.17; p = 0.044)	
Pre-REBOA SBP (mmHg)	0.96 (0.95–0.98; p < 0.001)	0.98 (0.96–1.00; p = 0.071)
Post-REBOA SBP (mmHg)	0.97 (0.97–0.98; p < 0.001)	0.98 (0.96–0.99; p = 0.006)

REBOA, resuscitative endovascular balloon occlusion of the aorta; cOR, crude odds ratio; aOR, adjusted odds ratio; SBP, systolic blood pressure; HR, heart rate; GCS, Glasgow coma scale; AIS, abbreviated injury scale; RBC, red blood cell; FAST, focused assessment with sonography in trauma.

### Model performance and validation

**Figure 3:**
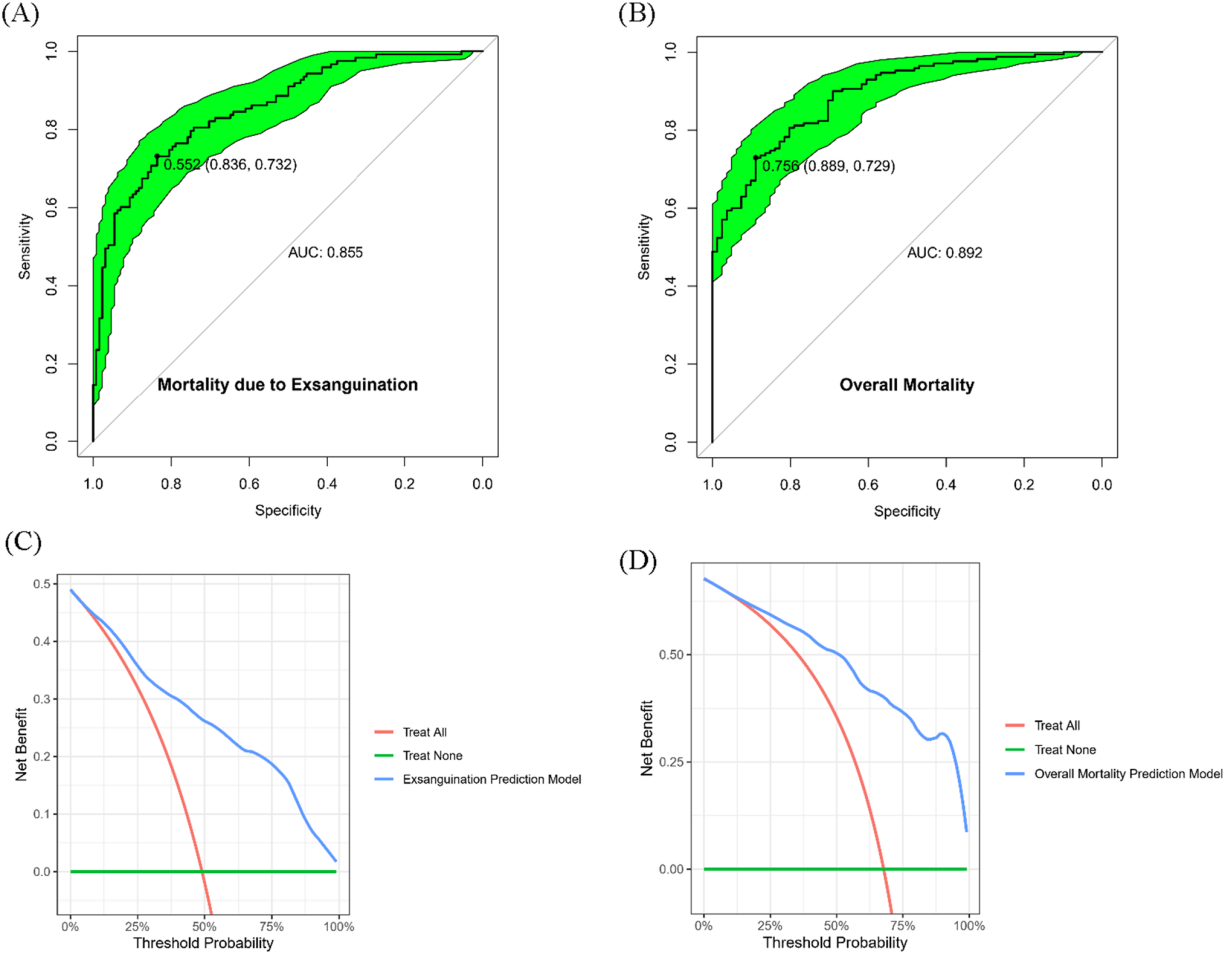
Accuracy of a multivariable logistic regression model for predicting mortality. (**A**) Mortality due to exsanguination. (0.855 AUROC, 0.552 probability as threshold, 0.836 specificity, and 0.732 sensitivity, respectively) (**B**) Overall mortality (0.892 AUROC, 0.755 probability as threshold, 0.889 specificity, and 0.756 sensitivity, respectively). Optimal cutoff value was presented using Youden’s index. 95% confidence interval was plotted by green. Decision curve analysis of (**C**) mortality due to exsanguination and (**D**) overall mortality.

The ROC curve and AUROC are presented in Fig. 3A,B, respectively. The decision curve analysis for the net benefit of each model is shown in Fig. 3C,D. The mortality due to exsanguination model showed an AUROC, sensitivity, specificity, positive predictive value (PPV), and negative predictive value (NPV) at the optimal threshold of 0.855, 73.2%, 83.6%, 81.1%, and 76.4%, respectively. The overall mortality model showed an AUROC, sensitivity, specificity, PPV, and NPV at the optimal threshold of 0.892, 72.9%, 88.9%, 93.2%, and 61.0%, respectively. Decision curve analysis revealed that the prediction model had greater net benefits than two extreme cases (all and no treatment). Of note, the net benefits of both models exhibited positive values across a wide range of threshold probabilities. The results for model validation using the bootstrap method are summarized in Supplementary Table 1. Index-corrected refers to the bootstrapped validated value. Index-original refers to Somers’ D of the original dataset. The training estimate is the average bootstrap model performance on the bootstrapped data. The test estimate is the average bootstrap model performance on the original unsampled data. Optimism, which refers to the difference between the training and test sets, was minimal in both models (0.0292 and 0.0235 in the mortality due to exsanguination and overall mortality models, respectively), indicating minimal overfitting. The calibration plot for each prediction model showed good consistency between the predicted and actual probabilities (Supplementary Fig. 1).

## Discussion

Our prediction models identified significant risk factors for mortality due to exsanguination and overall mortality using a novel nomogram that enables the calculation of each patient’s probability for mortality. Both prediction models showed favorable accuracy, with an AUROC of 0.855 and 0.892 for mortality due to exsanguination and overall mortality, respectively. The mortality due to exsanguination model identified five significant risk factors, namely initial HR, initial GCS, RBC transfusion within 4 h, balloon occlusion type, and post-REBOA SBP, whereas the overall mortality model identified four significant risk factors, namely initial GCS, RBC transfusion within 4 h, Young–Burgess classification, and post-REBOA SBP. Aside from the initial selection of patients, our model may provide useful information regarding the decision-making processes during or after REBOA. The intuitive nomogram can help clinicians make better decisions. Our model incorporated the patient’s response to REBOA such as post-REBOA SBP. This response of REBOA and nomogram could act as a warning for trauma surgeons, emphasizing the need for quicker and more proactive hemostatic measures. Indeed, the increasing risk and potential for medical futility should be considered. To the best of our knowledge, this has been the first study to propose a nomogram prediction model for mortality in trauma patients undergoing REBOA,which can be used for evaluating efficacy and response of REBOA. We anticipate that our nomogram will serve as a prognostic indicator.

The overall mortality and mortality due to exsanguination rates in the current study was 67.7% and 49.0%, respectively. Moreover, 48.2% of the included patients died within 24 h of admission, a figure that can be considered substantially high. A previous systematic review and meta-analysis regarding REBOA^4^ reported mortality rates of up to 75.9%. This high mortality rate might foster skepticism among certain clinicians^19^. Studies using propensity score matching based on national databases, such as the National Trauma Data Bank and Japan Trauma Data Bank, have reported unfavorable outcomes^20–22^. Indeed, a recent randomized controlled trial in the UK revealed that REBOA failed to demonstrate favorable outcomes^5^. Nonetheless, the utility of REBOA, including a new generation for partial REBOA, has been disseminated and regarded as a promising procedure for patients with exsanguination^23^. The first sole randomized controlled trial in the UK also has several limitations^24^. More relevant indications are warranted for the safe application of the REBOA procedure. The outcomes of our study could help resolve this issue. In our country, the REBOA kit is accessible at multiple level 1 trauma centers, and educational courses focusing on REBOA have been ongoing^25^. However, the rationale behind patient selection remains uncertain given that high mortality rates imply medical futility in certain patients. Indeed, estimating the exact intravascular volume status of exsanguinated patients is challenging^26,27^. Thus, our study may provide significant insights for health care providers. However, trauma surgeons should exercise caution when making decisions in cases with serious conditions, such as exsanguination. As shown in our decision curve analysis, a treat-all policy can yield significant net benefits. Considering the inherent adverse effects and invasiveness of REBOA, our prediction model can be useful given its increased net benefit even at elevated threshold probabilities, as demonstrated by our decision curve analysis^18^. The indications for REBOA in the current study are similar to those used by other trauma centers in the US^6^. However, the appropriateness of implementing a treat-all policy for patients with SBP below 90 mmHg remains controversial. The extremely high mortality rates observed herein suggests that REBOA may be futile for some patients. Our nomogram and decision curve analysis offer valuable insights regarding this issue.

Regarding risk factors for mortality, our study provides several significant insights. Previous studies on risk factors in patients undergoing REBOA have been limited. Hibert-Carius et al., in a retrospective study comprising 189 patients using the Aortic Balloon Occlusion (ABO) Trauma Registry from 22 centers in 13 countries, reported that the updated Revised Injury Severity Classification (RISC II) was the only risk factor for 30-day mortality on MLR analysis^28^. Yosuke et al., in a retrospective study comprising 207 patients from 23 hospitals across Japan, reported that ISS and time from arrival to arterial access were significantly associated with 30-day mortality^29^. They emphasized proactive arterial access based on their results. The current study did not use time-related variables considering the numerous missing values. In a retrospective study comprising 207 patients with pelvic fracture and Zone 3 REBOA from the Aortic Occlusion for Resuscitation in Trauma and Acute Care Surgery (AORTA) registry, Harfouche et al. reported that the GCS score was significantly associated with mortality. In our study, initial HR and GCS were significantly associated with mortality^30^. However, initial SBP was not identified as a significant risk factor, suggesting that the initial mental status appeared to be more significant than SBP. In another retrospective study comprising 524 patients using ABO Trauma Registry by the EVTM research group, Duchesne et al. reported that preinsertion SBP and delta SBP, defined as the difference between SBP prior to REBOA insertion and that after full aortic occlusion, were significantly associated with nonresponders who remained hypotensive with an SBP below 90 mmHg^31^. As such, they suggested that delta SBP could be a surrogate marker of hemorrhage volume and mortality. Similarly, the current study found that post-REBOA SBP, but not pre-REBOA SBP and delta SBP, was a significant risk factor for both overall mortality and exsanguination. This suggests that post-REBOA SBP, as a hemodynamic response after REBOA, appears to be a surrogate for mortality. Our study demonstrated that partial REBOA promoted more favorable outcome than did total occlusion. Recently, partial REBOA has attracted considerable attention given that one crucial limitation of REBOA is prolonged occlusion time, which can induce distal ischemia and consequent ischemia–reperfusion injury^10^. Although a systematic review of several clinical studies by Russo et al. reported promising results, more human studies are warranted^10^. Nonetheless, partial REBOA has been implemented in level 1 trauma centers across the US^6^, as well as in various level 1 trauma centers throughout South Korea. In our study, lateral compression pelvic fracture was a significant risk factor for overall mortality. Recent guidelines regarding pelvic fracture have considered not only fracture pattern but also hemodynamic status^32^. Notably, our cohort comprised hemodynamically unstable patients with pelvic fracture. Nonetheless, further studies are required regarding this issue. Our research demonstrated a significant association between the patterns of pelvic fractures and overall mortality rates, while failing to establish an association with exsanguination. Exsanguination seem to be related to hemodynamic status rather than pelvic fracture pattern. In contrast, pelvic fracture pattern may be related to other causes of mortality such as sepsis or multi-organ dysfunction.

The current study has several limitations worth noting. First, despite our inclusion of multiple centers including 251 patients, this study was retrospective in nature and may involve potentially substantial selection and survival biases. It cannot establish causality between risk factors and outcomes. We did not input transfusion within 24 h into the model given that numerous (48%) patients died within 24 h. Further prospective studies are warranted to estimate the exact effect size. Second, we enrolled consecutive patients starting from the first case in each center. We have no knowledge regarding the duration for which the plateau of the learning curve for the REBOA procedure would be reached. Knowledge and proficiency of REBOA may vary among trauma surgeons. Indeed, REBOA requires a multidisciplinary team approach, which would also be subject to a learning curve. This may affect prognosis, especially in the initial period. Third, some critical variables had numerous missing values, such as door-to-puncture time (33.9% missing), puncture-to-balloon time (34.3% missing), door-to-balloon time (5.6% missing), total occlusion time (19.9% missing), volume of ballooning (53.8% missing), and laboratory findings. Accordingly, these variables were excluded from the model. Fourth, partial REBOA was dependent on the tactile sense of the surgeon. Therefore, the actual blood flow passing through the occlusion site remains unclear. We did not use the new generation REBOA device (i.e., pREBOA-PRO™) for partial REBOA^33^. Furthermore, we did not use a distinct criterion for the application of partial REBOA. Consequently, the occurrence of both partial and total occlusion was an incidental outcome of the REBOA procedure rather than a premeditated strategic approach. Further studies are required to clarify this issue. Fifth, we did not use contraindications for REBOA, unlike several level 1 trauma centers in the US^6^. Patients with brain and chest traumas were included in our study. However, these injuries did not affect the model. Sixth, our model includes outcomes observed after the REBOA procedure, meaning that predictions are generated post-REBOA, rather than prior to performing the REBOA intervention. Consequently, our research focuses not on the indications for REBOA but on the prognosis following the REBOA procedure. Therefore, we included variables such as post-REBOA SBP, partial REBOA, and blood transfusion. Finally, we did not perform external validation. Although we performed bootstrap validation to overcome overfitting and obtained favorable results, the excellent performance of the prediction model may be attributed to overfitting. Nonetheless, further external validation studies are warranted.

## Conclusion

The novel nomogram prediction model proposed herein can accurately predict mortality due to exsanguination and overall mortality in severe trauma patients undergoing REBOA. Our model can be used as an intuitive tool for computing the likelihood of mortality for each patient, allowing speedy assessment of significant risk factors. Our prediction model revealed that total occlusion was associated with poor outcomes and that post-SBP could be an effective surrogate measure. The high risk indicated by our nomogram may serve as a warning signal. We believe that our model provides valuable insights, which would help trauma surgeons improve their decision-making process. Nonetheless, further prospective studies are warranted to estimate the exact effect size and overcome biases.

### Supplementary Information

Supplementary Information.

## Data Availability

The datasets used and/or analyzed during the current study available from the corresponding author on reasonable request.

## Abbreviations

REBOA: Resuscitative endovascular balloon occlusion of the aorta
IRB: Institutional review board
ISS: Injury severity score
AIS: Abbreviated injury scale
FAST: Focused assessment with sonography for trauma
SBP: Systolic blood pressure
IQR: Interquartile range
LASSO: Least absolute shrinkage and selection operator
MLR: Multivariable logistic regression
ROC: Receiver operator characteristic
AUROC: Area under the ROC curve
HR: Heart rate
GCS: Glasgow coma scale
RBC: Red blood cell
CI: Confidence interval
cOR: Crude odds ratio
aOR: Adjusted odds ratio
PPV: Positive predictive value
NPV: Negative predictive value
