# Supplementary material for: Nomogram for predicting in-hospital mortality in trauma patients undergoing resuscitative endovascular balloon occlusion of the aorta: a retrospective multicenter study
Source: Sci Rep. 2024 Apr 22;14:9164. doi: 10.1038/s41598-024-59861-3 (PMC11033263; doi:10.1038/s41598-024-59861-3)
Supplement: Supplementary file 1 — Supplementary Information. [file 41598_2024_59861_MOESM1_ESM.docx]

**Novel nomogram for predicting in-hospital mortality in trauma patients undergoing resuscitative endovascular balloon occlusion of the aorta: A retrospective multicenter study involving five Regional Trauma Centers across South Korea**


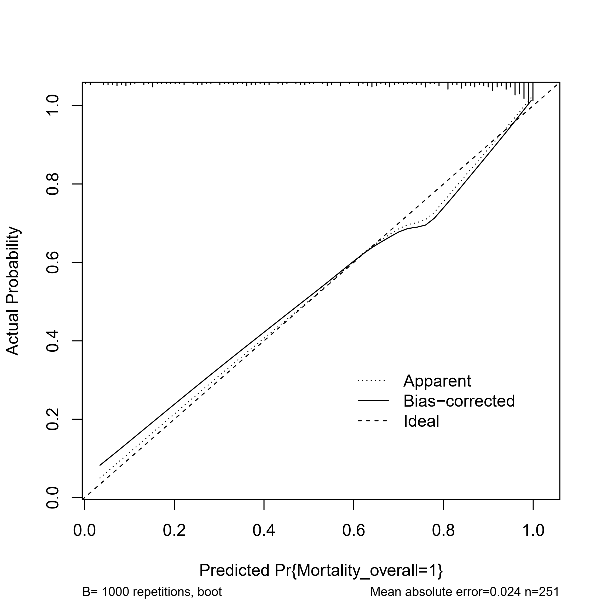

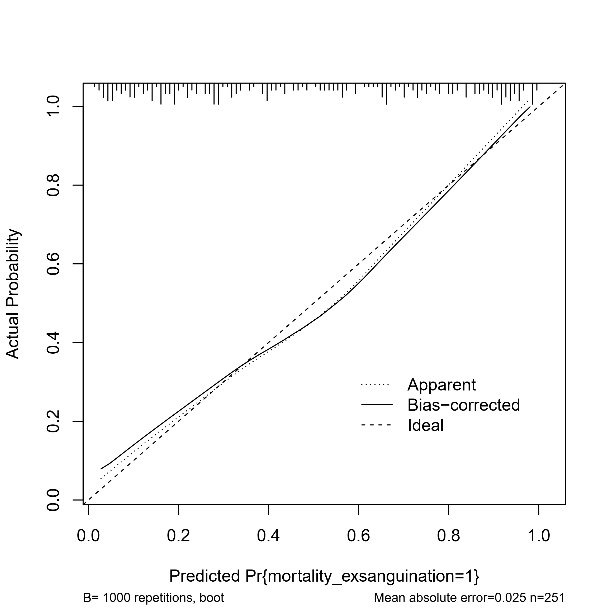
(A) (B)

**Supplementary Figure 1**. Calibration plot of (A) mortality due to exsanguination and (B) overall mortality.

| **Supplementary Table 1**. Bootstrap model validation (n = 1000) | | | | | | | |
| --- | --- | --- | --- | --- | --- | --- | --- |
| **Model** | **Metric** | **Index-original** | **Training** | **Test** | **Optimism** | **Index-corrected** | **n** |
| Mortality due to exsanguination | Dxy | 0.7105 | 0.7267 | 0.6975 | 0.0292 | 0.6813 | 1000 |
| Overall mortality | Dxy | 0.7830 | 0.7956 | 0.7722 | 0.0235 | 0.7595 | 1000 |
| Dxy, Somers’ D; Index-original, original estimate of Dxy; Training, average bootstrap model performance; Test, average bootstrap model performance on the original unsampled data; Index-corrected, Optimism, difference between training and test; bootstrapped validated value | | | | | | | |
